# The impact of predisposing, enabling, and need factors in utilization of health services among rural residents in Guangxi, China

**DOI:** 10.1186/s12913-016-1825-4

**Published:** 2016-10-19

**Authors:** Yan-Ning Li, Dong-xiao Nong, Bo Wei, Qi-Ming Feng, Hong-ye Luo

**Affiliations:** 1 Department of Health Service Management, Guangxi Medical University, Nanning, Guangxi People’s Republic of China; 2 The First Affiliated Hospital of Guangxi Medical University, Nanning, Guangxi People’s Republic of China

**Keywords:** Andersen behavioral model, Health service utilization, Impact

## Abstract

**Background:**

Healthcare in China has significantly improved, meanwhile many  socio-economic risk factors and health conditions factors affect accessibility and utilization of health services in rural areas. Inequity of health service in China needs to  be estimated and reduced. Andersen behavioral model is useful to assess the association of health service utilization with predisposing, enabling, and need factors.

**Methods:**

A survey was conducted among 4634 residents of 897 households in 2012. Logistic regression analysis was performed to explore the association of predisposing (age, gender, marital status, ethnicity and family size), enabling (education level, travel time to the nearest health facility, medical expense per capita, and health insurance coverage), and need factors (chronic disease) with the utilization of health services (i.e. physician visit and hospitalization).

**Results:**

We observed a significant association between need factor (chronic diseases) and health service unitization, after adjusting for all predisposing and enabling factors (physician visits: odds ratio (OR) = 5.87, 95 % confidence interval (CI) = 4.71–7.32; hospitalization: OR = 4.04, 95 % CI = 2.90–5.61, respectively). In addition, age, gender, marital status, family size and education level were significant predictors of health service utilization. The travel time to the nearest health facility was associated with the utilization of physician visits, and expenditure on healthcare was a hindering factor of hospitalization.

**Conclusions:**

The predisposing and enabling factors had a minor impact on health service utilization, while the need factor was a dominant predictor of health service utilization among rural residents in China.

**Electronic supplementary material:**

The online version of this article (doi:10.1186/s12913-016-1825-4) contains supplementary material, which is available to authorized users.

## Background

Healthcare in China has significantly improved with rapid economic development. At the same time, the cost of health services and health care expenditures keep rising rapidly by the widely use of high-tech medical devices and costly drugs. The assessment of access to health services includes geographical accessibility, financial accessibility, availability and acceptability [1]. These factors are complicated in a large developing country, therefore, access to health service is becoming increasingly unequal in China, particularly in rural areas. Health policies, especially government investments and the cost of health service, vary significantly in different regions [2]. A new governmental public health service funding for rural areas was started in 2010. However, the funding for economically developing rural areas is still not so sufficient as that in economically developed rural areas [3]. The gap between the wealthy and poor keeps widening despite of the significant overall economic improvement of rural residents in the past 30 years. The gap, owing to geographic and economic factors, potentially affects accessibility and utilization of available health services [4].

The National Health Service Survey (NHSS) in 2009 shows that the patients, should have been hospitalized, are 2.1 times in the poorest groups as those in the richest group [5]. Basic health services, lacking sufficient equipment and staff, are common in remote rural areas of China. Most government programs to improve health service, e.g. New Cooperative Medical Scheme (NCMS) in rural China, focus on helping rural residents. NCMS, providing overall coverage, is a primary medical security system for rural China in order to improve access to health services and prevent impoverishment due to medical expenses. In this system, personal contributions to the insurance premium are relatively low with allowances from the local and central government. According to the Poverty Monitoring Report on Rural China 2010, the percentage of Chinese peasant unable to receive appropriate health services for financial reasons dropped by 7.8 % from 2002 to 2009 [6]. NCMS has increased the use of healthcare services, while NCMS reimbursements are mainly provided for inpatient expenses.

Despite the situation that the inequities of health service utilization in China are increasing [7, 8], there is little literature concerning socio-economic risk factors and health conditions in rural areas. Over the past two decades, sociologists and epidemiologists emphasize that studies on health services utilization help understanding influence factors and guide policy strategy to improve health service delivery.

The Andersen health behavior model [9] is widely accepted as a reliable tool for the study of health services utilization. According to the Andersen model, health service utilization is a sequential and conditional function of three sets of factors: predisposing (demographic and social) factors, enabling (economic) factors, and need (health outcomes) factors. Predisposing factors reflect the individuals’ propensity to use health services, enabling factors are the resources that may facilitate access to services, and the need factors represent potential needs of health service use, such as self-perceived health, chronic conditions, and restricted activity [10].

The Andersen model has been revised to account for not only medical care but also the individual. Studies showed that, based on the Andersen behavioral model, equity in health service is achieved when the need factors have a strong positive association with health service utilization [11]. On the other hand, enabling resources (e.g. health insurance or income) may lead to inequity in health service [12]. Need of health service may be affected by other socio-economic factors, such as ethnicity [13]. Arcury *et al*. considered that preventive care utilization would mostly be influenced by predisposing and enabling factors, while curative care and hospitalization would primarily be influenced by need factors [14].

NHSS is a national population survey, which was conducted by the Center for Health Statistics and Information in collaboration with the Ministry of Health of China. It supported the drafting of rural health policies facing local development, the difficulty of access to health service and high expenditures. Moreover, the survey aimed to summarize the present situation of health services, influencing factors in rural areas, as well as to allocate health resources reasonably.

An investigation, according to the survey method and content of NHSS, was conducted in rural Guangxi. Besides the assessment of health service utilization in poor areas, the Andersen model was applied to study the influencing factors associated with health service utilization in rural individuals. The results hopefully provide potential advice to health policy by addressing health service needs in poor areas and the utilization of services.

## Methods

### Study setting and participants

China was geographically grouped into urban (i.e., cities) and rural (i.e., town or villages) areas. China’s *per capita* gross domestic product (GDP) was RMB 38354 (approximately US$6100) in 2012, and the average net income for farmers was RMB 7919 (approximately US$1250) [15].

Guangxi is an economically developing agricultural province in western China. The population was estimated at 52,820,000 inhabitants in 2012. The *per capita* GDP in Guangxi was RMB 30,588 (approximately US$4829), and the average net income of farmers was RMB 6213 (approximately US$981) [16]. Guangxi's income level was lower than the national average, especially in rural areas. This study focuses on Guangxi, one of typical developing provinces, to investigate the need of health service in rural China.

Guangxi province has more public health problems compared to developed provinces in China. Low government health expenditures and inadequate health resources is a common problem in these developing regions [17]. Most poor people in Guangxi are villagers in remote and mountainous regions with low levels of education and weak awareness of health protection, all of which are susceptible risks of the spread of infectious diseases [18].

The eligible participants in this study were the Guangxi peasants living in the sampling rural area for not less than 6 months. The participant was excluded if three investigation failures had happened or unable to communicate (verbally or written). The recall bias of self-reported prevalence is inevitable in a retrospective investigation. To reduce the bias, the survey required participants to recall illness during the last two weeks of the survey in accordance with international practice.

A cross-sectional study, using multi-stage stratified cluster random sampling for household survey (county and township cluster, and simple random sampling of households), was carried out from June 30 to July 20, 2012. The single proportion formula of the sample size was 1.96^2^ *pq (DEFF)*/*d* ^*2*^ (*p* = two-week prevalence of 17 % in rural Guangxi [19], *q* = 1 − p and *d* = margin of error of 1.5 %). The adjusted sample size was 4818 by multiplying a factor of 2 due to the design of multi stage sampling [20]. Considering a possible 10 % non-response rate, the final sample size was 5300.

Counties were stratified into two groups (above or equal to average, below the average), based on *per capita* GDP of counties in Guangxi [21]. Two counties, Rongxian County (above the average *per capita* GDP) and Luchuan County (below the average *per capita* GDP), were chosen. Three townships were selected in each county. Three villages were selected in each township, based on population size. Households were randomly selected from sample villages and all family members in a sampled household were individually interviewed.

The survey interviewed each household member in the sample families. The interviewers were trained students from the public health school of Guangxi medical university. The parents were asked to provide relevant information in cases of children younger than 15 years old.

### Variable content

In the household health interview, a questionnaire was used for data collection (Additional file 1). The main contents of the questionnaire were health service utilization in household members. It included characteristics of socio-economic status, demographics, and insurance for the residents; self-reported illness and 2-week prevalence prior to the survey; consultation, hospitalization, and expenditure of both inpatient and outpatient.

Predisposing factors were sociological factors, such as age (years), gender (male, female), ethnicity (Han, minority), marital status (married, single, separated/divorced/widowed), and family size (number of family members). Enabling factors deserve much attention because they explain barriers and facilitators to service use, which in turn can be targets of interventions. Educational level (as quintile, 1 = no education to 5 = college or more, “no education” means “has never gone to school”) and accessibility of health service (shortest travel time to nearest health facility, *per capita* expenditure on healthcare, expenditure of NCMS) were considered enabling factors. Need factors are overall health condition, as indicated by chronic illness or not. For chronic conditions (e.g., arthritis, stroke, cardiac problems, diabetes mellitus, cancer), individuals were asked to report, using a yes-or-no format, existing illnesses or symptoms with at least 3 months continuous or intermittent presentation within the last 6 months.

The outcome of healthcare utilization is measured in two perspectives: the utilization of outpatient visit (e.g., physician visit) and the utilization of inpatient visit (e.g., hospitalization). The utilization of outpatient visit refers to the questionnaire item “have you been to a clinic in the last two weeks?” The utilization of inpatient visit is based on the questionnaire item “Have you been hospitalized in the past half year?” These two variables were dichotomous (yes or no).

### Statistical analysis

The database was created in Epidata 3.0. Statistical power was calculated with PASS version 11.0. Other analyses were performed using SPSS version 17.0. Descriptive statistics were used to describe the social, economic, and demographic characteristics of rural residents. To test the statistical significance of the difference between the various subgroups in the percentage of reported problems, χ^2^ tests and z tests were used. Subsequently, multivariate logistic regressions were used to determine the association of health service utilization with the predisposing, enabling, and need characteristics. Multivariate analyses were performed in three different steps (i.e., 1. considering the need factor; 2. inserting the predisposing characteristics; and 3. considering the enabling resources), which allowed the assessment of the influence of need, controlling for possible confounding associations on health service utilization. Statistical significance was considered when two-sided P ≤ 0.05, and the results were expressed as odds ratios (OR) with 95 % confidence intervals (CI).

## Results

### Sociodemographic characteristics in rural residents in Guangxi

The questionnaire survey was conducted in 5300 potentially eligible individuals, out of which non-eligible people included 98 non-reachable for three attempts and 3 unable to communicate in mandarin. The participant rate was 98.09 % (5199/5300). Among the 5199 eligibility, 2 refused to cooperate with the interview due to privacy. The rest of 5197 participants completed the questionnaire with the correct participant rate of 98.06 % (5197/5300). Missing answers to the key questions, such as inpatient utilization and chronic diseases, were found in 563 participants, the rest 4634 valid subjects were analyzed in this study. The correct response rate was 87.43 % (4634/5300). The statistical power was 0.987 indicating reliable results.

In the 4634 subjects, 686 (14.80 %) subjects had used outpatient service in the last two weeks, and 280 (6.04 %) subjects had used inpatient service in the last six months. Descriptive analysis by socio-economic status is shown in Table 1. The sample consisted of 4634 respondents with an average age of 32.72 ± 21.76. More than half (52.42 %) were male, and 53.69 % were married. The average family size was 6.02 people. An educational level below junior middle school was reported by 48.70 % of the respondents. The average travel time to the nearest health facility was 10.52 min. The mean expenditure on healthcare per year was RMB 4242.16, 98.2 % covered by national health insurance with a cost of RMB 172.92 per year. There were 11.57 % of the subjects suffered from chronic diseases.

**Table 1:** Descriptive information of study subjects

Characteristics	Total number	Value/percentage
*Predisposing factors*		
Age (in years)	4634	32.72 ± 21.76
Gender		
Male	2429	52.42 %
Female	2205	47.58 %
Marital status		
Single	1973	42.58 %
Married	2488	53.69 %
Separated/Divorced	18	0.39 %
Widowed	155	3.34 %
Ethnicity		
Han	4583	98.90 %
Others	51	1.10 %
Family size	4634	6.02 ± 2.26
*Enabling factors*		
Educational level		
No education	905	19.52 %
Primary school	1352	29.18 %
Secondary school	1784	38.50 %
High school	457	9.86 %
College or more	136	2.93 %
Time to nearest health facility (min)	4634	10.52 ± 11.29
Medical expense *per capita* (RMB)	4634	4242.16 ± 9046.85
Health insurance coverage (RMB)	4634	172.92 ± 69.40
*Need factors*		
Chronic diseases		
Yes	536	11.57 %
No	4098	88.43 %

### Potential risk factors for the utilization of physician visits and hospitalization

As one of the predisposing factors, age was positively associated with the utilization of physician visits (Table 2) and hospitalization (Table 3). Females and married people showed health seeking behaviors similar to the age factor. However, family size was negatively associated with the utilization of outpatient. As enabling factors, the travel time to health facility and expenditure on NCMS were associated with the utilization of physician visits, but not hospitalization. Conversely, expenditure on healthcare for inpatient but not outpatient was significant. Educational level was negative associated with the two utilizations. For the need factor, subjects with chronic diseases tended to use physician visits and hospitalization more than those without chronic diseases.

**Table 2:** Potential risk factors for the utilization of outpatient services

Characteristics	Physician visit	No physician visit	*P* value
	(*n* = 686)	(*n* = 3948)	
*Predisposing factors*			
Age (years)	41.51 ± 26.29	31.19 ± 20.50	<0.001
Gender			
Male	313	2116(14.79 %)	<0.001
Female^a^	373	1832(20.36 %)	
Marital status			
Single^a^	208	1765(11.78 %)	<0.001
Married	432	2056(21.01 %)	
Separated/Divorced/Widowed	46	127(36.22 %)	
Ethnicity			
Han	672	3911(17.18 %)	0.011
Others^a^	14	37(37.84 %)	
Family size (people)	5.56 ± 2.25	6.10 ± 2.25	<0.001
*Enabling factors*			
Educational level			
No education^a^	219	686(31.92 %)	<0.001
Primary school	245	1107(22.13 %)	
Secondary school	183	1601(11.43 %)	
High school	36	421(8.55 %)	
College or more	3	133(2.26 %)	
Time to nearest health facility (min)	10.21 ± 10.60	12.35 ± 14.71	<0.001
Expenditure on healthcare (RMB)	4671.77 ± 7433.72	4167.51 ± 9297.44	0.180
Expenditure of NCMS (RMB)	159.31 ± 67.05	175.29 ± 69.53	< 0.001
*Need factors*			
Chronic diseases			
Yes	246	290(84.83 %)	<0.001
No^a^	440	3658(12.03 %)	

^a^ reference group

**Table 3:** Potential risk factors for the utilization of inpatient services

Characteristics	Hospitalization	No Hospitalization	*P* value
	(*n* = 280)	(*n* = 4354)	
*Predisposing factors*			
Age (years)	40.92 ± 25.07	32.19 ± 21.43	< 0.001
Gender			
Male	113	2316(4.88 %)	< 0.001
Female^a^	167	2038(8.19 %)	
Marital status			
Single^a^	54	1919(2.81 %)	< 0.001
Married	205	2283(8.89 %)	
Separated/Divorced/Widowed	21	152(13.82 %)	
Ethnicity			
Han	277	4306(6.43 %)	0.960
Others^a^	3	48(6.25 %)	
Family size (people)	6.02 ± 2.45	6.02 ± 2.24	0.980
*Enabling factors*			
Educational level			
No education^a^	82	823(9.96 %)	< 0.001
Primary school	65	1287(5.05 %)	
Secondary school	109	1675(6.51 %)	
High school	21	436(4.82 %)	
College or more	3	133(2.26 %)	
Time to nearest health facility (min)	9.94 ± 9.36	10.56 ± 11.41	0.370
Expenditure on healthcare (RMB)	8323.18 ± 16336.27	3979.71 ± 8298.49	< 0.001
Expenditure of NCMS (RMB)	170.43 ± 75.47	173.08 ± 68.99	0.540
*Need factors*			
Chronic diseases			
Yes	96	440(21.82 %)	< 0.001
No^a^	184	3914(4.70 %)	

^a^ reference group

### Multivariate analysis for the association between the utilization of health services and its potential risk factors

Table 4 summarizes a series of multiple logistic regression analyses to estimate the relationship between the health services utilization and the predisposing, enabling, and need factors. For the physician visit, there was a strong positive association between self-reported health problems and the utilization. In the unadjusted model, the need factor entered the initial model making a significant contribution. Those who had chronic diseases were more willing to use outpatient and inpatient health service. After adjusting all predisposing and enabling factors, the influences of chronic diseases were still observed (physician visits: OR = 5.87, 95 % CI = 4.71–7.32; hospitalization: OR = 4.04, 95 % CI = 2.90–5.61, respectively). In addition, the age factor was significantly associated with hospitalization but not physician visits. Married people had greater OR compared to singles. People with high education levels were less likely to use health services.

**Table 4:** The odds ratio and 95 % CI from multivariate logistic regression models for the utilization of physician visit and hospitalization

Characteristics	Physician visit		Hospitalization	
	OR (95 % CI)	*P* value	OR (95 % CI)	*P* value
*Predisposing factors*				
Age (year)	1.00 (0.99–1.00)	0.280	1.02(1.01–1.03)	< 0.001
Gender	1.20(1.01–1.43)	0.040	1.40(1.08–1.81)	0.010
Marital status				
Single ^a^				< 0.001
Married	1.50 (1.22–1.85)	< 0.001	5.30 (3.28–8.55)	
Separated/Divorced/Widowed	0.99 (0.70–1.54)	0.970	7.06 (3.26–15.26)	< 0.001
Ethnicity	1.03 (0.51–1.21)	0.270	1.59 (0.63–4.06)	0.330
Family size (people)	0.92 (0.88–0.96)	<0.001	0.94 (0.84–1.06)	0.940
*Enabling factors*				
Educational level	0.58 (0.52–0.64)	< 0.001	0.75 (0.65–0.86)	< 0.001
Time to nearest health facility (min)	0.90 (0.81–0.92)	< 0.001	0.98 (0.995–1.019)	0.240
Expenditure on healthcare (RMB)	1.00(0.991–1.001)	0.610	1.01(1.002–1.099)	< 0.001
Expenditure of NCMS (RMB)	1.00(0.998–1.004)	0.450	1.00(0.999–1.006)	0.190
*Need factors*				
Chronic diseases	5.87 (4.71–7.32)	< 0.001	4.04 (2.90–5.61)	< 0.001
Yes				
No				

^a^ reference group

### Medical expenditures of health services in the rural area and the whole Guangxi province

For the 536 subjects with chronic diseases, only 246 (45.90 %) reported physician visits, and only 96 (17.91 %) reported being hospitalized. The commonest reported reason for not using a health service even with chronic diseases was financial difficulty.

Table 5 shows medical expenditures, payments, and proportions in rural areas and the entire province of Guangxi. The medical expenditure per physician visit in rural areas of Guangxi was higher than that for the entire province of Guangxi, while the mean hospitalization expenditure in rural areas (RMB 2507) was much lower than that of the entire province (RMB 3648, of which RMB 976 could be reimbursed) (Table 5). The reimbursement proportions were 26.8 % (976/3648) in the entire province and 30.4 % (762/2507) in the rural areas.

**Table 5:** The medical expenditure, payment, and proportion in the rural area and the entire province of Guangxi

Medical expenditure and payment	Guangxi province	Rural area in Guangxi^a^
Medical expenditure per physician visit (RMB)	86.7	99.7
Proportion of the payment of clinic expenses (%)		
NCMS account	20.7	28.8
Reimburse partly or deduction	4.9	12.4
Self-paid	74.4	58.8
Mean expenditure of hospitalization (RMB)	3648	2507
Reimbursement of hospitalization (RMB)	976	762

^a^ The data come from Household Health Service Investigation in Guangxi 2008

## Discussion

This study assessed 10 predisposing, enabling, and need factors associated with the utilization of health services in a rural setting in Guangxi, China. The strength of our study lies in the conceptual framework based on Andersen’s health behavior model. We found that the need factor (chronic diseases) was the dominant predictor of health service utilization. The predisposing and enabling factors affected the utilization to some extent.

### Predisposing factors

We observed that the predisposing factors, including age, gender, family size and marital statue contributed significantly to the variance in health service utilization. Numerous studies showed a positive association between socio-economic status and health in Europe [22, 23], as well as in China’s previous studies [24, 25]. Among all predisposing factors, marital status had a stronger effect than age, gender and family size, which is consistent with previous studies [26, 27]. As marital status affects health seeking behavior, a possible explanation is that married people get help and advice from their spouse promoting a doctor visit. Younger individuals were found more likely to use inpatient health services, possibly due to parental attention to young children. In this study, the OR of gender was 1.2 for physician visits and 1.4 for hospitalization, indicating that females have more health needs and awareness than males. Gender equality is an important issue in health service utilization. Many studies showed the differences in health seeking behavior between males and females [28, 29]. In addition, the family size was negatively associated with the utilization of health services, in another word, smaller family size linked to more health service utilization. These data suggested that married females with smaller family sizes were more likely to endorse willingness to use health services. These people should be a key target group for health interventions.

### Enabling factors

Educational level was negatively associated with the health service utilization, which is consistent with several previous studies [17]. Higher education may lead to more knowledge of preventive health care and awareness of health services. For this population, it is important to improve their personal ability of health control by influencing their lifestyles and to increase the use of health services [30]. Travel time to the health facility, consistent with the previous findings [31, 32], was negatively associated with the utilization of physician visit. Therefore, more village clinics should be strategically set up to guarantee convenience in access to primary health services. The reason for significant personal expenditures associated with the hospitalization utilization may be the result of the present health policies, hospitalization means more out-of-pocket expenditures compared to outpatient services. Association between the expenditure of NCMS and the utilization of health services was not found, reflecting the benefits from the popularized China rural basic medical insurance. According to China health statistics yearbook, the scheme covered 832 million rural residents, or 97.5 % of Chinese farmers, and the government contribution to insurance premium increased to 246 RMB (US$38.51) per person in 2011 [33].

### Need factor

Chronic conditions represent potential needs for healthcare use. Our findings are comparable to other studies showing patients with chronic illness were more likely to use outpatient and inpatient services [34–36]. These suggest that chronic diseases play a critical role in increasing health service utilization by promoting a more positive personal perception.

As mentioned above, many socio-economic characteristics play an important role in the utilization of health services in the process of seeking medical attention of rural residents. These factors affect their decisions and thereby affect the choice of medical service expenses. Primary health care institutions are of great importance in the rural health service system, village clinics serve as basic health maintenance, and county-level hospitals are the main choice for the treatment of serious disease. In this study, most outpatient service happened in village clinics and over a half of inpatient service happened in county-level hospitals. Thus, enhancing village clinics and county-level hospitals, including improving financial investment, infrastructure and staff training, is necessary for better access to the rural primary health care.

In rural areas of China, personal or family economic difficulty is a major problem, particularly in case of hospitalization. The reimbursement proportions in both this study and Guangxi province are low. Residents in rural areas must pay for the rest of medical expenditure, which still leads to a heavy economic burden. Since 1990, the increasing use of high-level medical technologies and expensive drugs has raised up expenditures on health service in China. Studies also showed that the health expenditure *per capita* has grown since the 1980s [37]. Therefore, many people with low income have experienced increased financial difficulty in seeking health services [38]. It is necessary to cut down medical expenditures in rural areas to relieve the economic burden. NCMS sets deductibles and a ceiling, poor residents might be consequently out of the coverage due to financial difficulties. Therefore, NCMS is a basic guarantee for health service and other supplemental insurances are needed.

### Study limitations

This study has some limitations. Self-reported responses used in this investigation is a common way to evaluate the use of health services [39, 40]. Reporting bias is a major disadvantage in the assessment of health care because of self-reported data [41]. The recall period in health surveys, 2 weeks for outpatient and 6 months for inpatient, is accepted for eliminating the error. Clark et al. [42] found that the recall accuracy is positive associated with hospitalization time. Clarke et al. [39] reported recall period should be determined in accordance with survey technique, the outcome, and policy. The estimated sample size was 5300, while the subjects of analysis were 4634. Non-response is a source of missing data. There are different non-response bias effects, and non-response on disease contributes most to the bias [43]. The dependent variable in the regression was a simple categorical variable. Further studies need to conduct continuous variables and items to expand the basic knowledge and improve the attitudes in rural adults toward health services. Despite these limitations, the present study collected important information on health service in Chinese rural adults. Furthermore, our findings may provide baseline information for educational interventions to promote healthcare utilization awareness in rural populations.

## Conclusions

The results showed the impact of economic status, health status, demographic and social characteristics, the medical insurance system, and other factors on the health service utilization of rural residents. Chronic disease, representing the need factor in the Andersen model, was a dominant predictor of health service use. The findings from this study may inform policy makers of the need of equitable use of rural health services and lead to detailed policy-directed researches.

## Additional file

Additional file 1: The survey queationnair of health service. (DOCX 25 kb)
