# Supplementary material for: The impact of predisposing, enabling, and need factors in utilization of health services among rural residents in Guangxi, China
Source: BMC Health Serv Res. 2016 Oct 19;16:592. doi: 10.1186/s12913-016-1825-4 (PMC5070132; doi:10.1186/s12913-016-1825-4)
Supplement: Additional file 1: — The survey queationnair of health service. (DOCX 25 kb) [file 12913_2016_1825_MOESM1_ESM.docx]

**THE HEALTH SERVICE SURVEY** **IN RURAL AREA**

**Householder Name____________**

**Address: ___________County(City/District)____________ Town(Street)**

**____________ Village________________________**

**Zip Code □□□□□□**

**Town Code □ Village Code □ Resident Code □□□**

**Finish Date** **____/___/___ Surveyor Signature****___________**

**Check Date ____/___/___ Inspector Signature___________**

**Table 1. Family General** **Condition**

| **NO.** | **Question&Option** | **Answer** |
| --- | --- | --- |
| 1 | How many people are there in your family? (including household population, relatives or nannies who lived together in half a year ) |  |
| 2 | How much time does it take to get the nearest health facility from your home? (min) (the most convenient way, such as by public transport or on foot ) |  |
| 3 | Which medical facility is closest to your home?  (1) Private clinic (2) Public clinic or community health station (3) Township hospital/ community health center  (4) County /District general hospital (5) County /District Chinese medicine hospital (6) City hospital  (7) Provincial hospital (8) Others |  |
| 4 | How much money did your family spend on living consumption last year? (YUAN) |  |
| 5 | Out of the total consumption, how much was spent on medicines, medical supplies and health services? (YUAN) |  |
| 6 | Did your family participate in New Cooperative Medical Scheme? (1) yes (2) no (3) once but quit |  |
| 7 | How much did your family pay for New Cooperative Medical Scheme last year? (YUAN) |  |

**Table 2. Health Condition of Each Family Members**

| **Respondents’ code (Householder is NO.01, other members by order )** | | **01** | **02** | **03** | **04** | **05** | **06** |
| --- | --- | --- | --- | --- | --- | --- | --- |
| **A. Demographic characteristics** | |  |  |  |  |  |  |
| 1 | Name: |  |  |  |  |  |  |
| 2 | Relationship with householder: (1) Householder (2) Spouse (3) Child (4) Grandchild  (5) Parent (6) Grandparent (7) Sibling (8) Others |  |  |  |  |  |  |
| 3 | Gender: (1) Male (2) Female |  |  |  |  |  |  |
| 4 | Ethnicity: (1) Han (2) Others |  |  |  |  |  |  |
| 5 | Age (years): |  |  |  |  |  |  |
| 6 | Marital status: (1) Single (2) Married (3) Separated/Divorced (4) Widowed |  |  |  |  |  |  |
| 7 | Education level: (1) No education (2) Primary school (3) Secondary school  (4) High school (5) College or more |  |  |  |  |  |  |
| 8 | Which medical facility did you use most?  (1) Private clinic (2) Public clinic or community health station  (3) Township hospital/ community health center (4) County /District general hospital  (5) County /District Chinese medicine hospital (6) City hospital  (7) Provincial hospital (8) Others |  |  |  |  |  |  |
| 9 | What is the reason for the answer to No.8?   1. Short distance (2) Fair price (3) High level medical technology   (4) Advanced medical equipment (5) Abundant drugs (6) Excellent service  (7) Insurance coverage (8) Familiar stuff (9) Reliable doctors (10) Others |  |  |  |  |  |  |
|  | | | | | | | |

| **B. Prevalence** | | | | | | | |
| --- | --- | --- | --- | --- | --- | --- | --- |
| 1 | Have you been to a clinic in the last two weeks? (1)Yes (2) No |  |  |  |  |  |  |
| 2 | Have you ever been diagnosed with chronic diseases? (1)Yes (2) No |  |  |  |  |  |  |
| 3 | (1)Disease (If there are multiple chronic diseases, please fill in sequence ) |  |  |  |  |  |  |
| 4 | First disease code: |  |  |  |  |  |  |
| 5 | Diagnosis time: (1)six months ago (2)within six months |  |  |  |  |  |  |
| 6 | Have the disease been treated in six months? (1)Yes (2)No |  |  |  |  |  |  |
| 7 | If be treated, how much money did you spend every month? |  |  |  |  |  |  |
| 8 | If not be treated, the main reason: (1) Feel mild (2) Inconvenient traffic  (3) Economic difficulties (4) Treatment dose not work (5) Others |  |  |  |  |  |  |
| 9 | (2) The second disease |  |  |  |  |  |  |
| 10 | Second disease code: |  |  |  |  |  |  |
| 11 | Diagnosis time: (1)six months ago (2)within six months |  |  |  |  |  |  |
| 12 | Have the disease been treated in six months? (1)Yes (2)No |  |  |  |  |  |  |
| 13 | If be treated, how much money did you spend every month? |  |  |  |  |  |  |
| 14 | If not be treated, the main reason: (1) Feel mild (2) Inconvenient traffic  (3) Economic difficulties (4) Treatment dose not work (5) Others |  |  |  |  |  |  |
| 15 | Have you been hospitalized in the past half year? (1)Yes (2)No (jump to No.17) |  |  |  |  |  |  |
| 16 | How many times you have been hospitalized in the past half year? |  |  |  |  |  |  |
| 17 | The reason for no hospitalization: (1) Unnecessary (2) Treatment dose not work  (3) Economic difficulties (4) Bad service  (5) Busy (6) Unavailable hospital beds (7) Others |  |  |  |  |  |  |
